# Beyond diabetes and obesity: GLP-1 receptor agonists as multifunctional therapeutics across the steatotic liver disease spectrum

**DOI:** 10.3389/fphar.2026.1752204

**Published:** 2026-05-15

**Authors:** Sundararajan Mahalingam, Kusum K. Kharbanda, Carol A. Casey, Karuna Rasineni

**Affiliations:** 1 Research Service, Veterans Affairs Nebraska-Western Iowa Healthcare System, Omaha, NE, United States; 2 Department of Biochemistry and Molecular Biology, University of Nebraska Medical Center, Omaha, NE, United States; 3 Department of Internal Medicine, University of Nebraska Medical Center, Omaha, NE, United States

**Keywords:** alcohol-associated liver disease (ALD), glucagon-like peptide 1 receptor agonists (GLP-1 RAs), metabolic dysfunction and alcohol-associated liver disease (MetALD), metabolic dysfunction-associated steatotic liver disease (MASLD), steatotic liver disease (SLD)

## Abstract

The global incidence of steatotic liver disease (SLD), driven by metabolic dysfunction-associated steatotic liver disease (MASLD), alcohol-associated liver disease (ALD), and the synergistic metabolic dysfunction and alcohol-associated liver disease (MetALD), is fueling a rapid rise in hepatocellular carcinoma (HCC). Regardless of the initial etiology (metabolic, alcoholic, or viral), progression to advanced SLD and HCC is governed by critical shared pathways, which are systemic metabolic dysregulation and inflammation. Identifying a single, targeted pharmacological agent to address this unified pathophysiology is an urgent unmet need. This review addresses the epidemiological links between SLD etiologies and HCC, dissecting their shared metabolic pathophysiology. We evaluate the emerging potential of glucagon-like peptide-1 receptor agonists (GLP-1 RAs) as a multifunctional therapeutic strategy to target this metabolic hepatic nexus. GLP-1 RAs offer a dual central and peripheral mechanism against SLD progression. Centrally, these GLP-1 RAs modulate appetite and reduce the intake and cravings for high-calorie food and alcohol. Peripherally, these agents induce significant weight loss, enhance insulin sensitivity, and reduce hepatic *de novo* lipogenesis. While their efficacy in resolving metabolic dysfunction-associated steatohepatitis (MASH) is increasingly well documented, their ability to target the metabolic hepatic nexus also suggests a promising therapeutic role in ALD and MetALD. Furthermore, GLP-1 RAs appear to exert direct anti-inflammatory and anticancer effects by activating metabolic sensors, such as the AMPK pathway, to inhibit proliferative signaling. Clinical and preclinical data support the efficacy of GLP-1 RAs in resolving steatosis and SLD progression in metabolic contexts. By targeting shared metabolic dysregulation, GLP-1 RAs emerge as potential candidates for HCC risk mitigation and may serve as future therapeutic adjuvants. Future large-scale, prospective clinical trials are warranted to confirm these benefits, particularly in ALD and MetALD, where underlying metabolic dysfunction remains a significant driver of disease progression.

## 1 Introduction

The global burden of steatotic liver disease (SLD), also known as fatty liver disease, is undergoing a profound epidemiological shift. It is rapidly moving away from viral hepatitis as the sole primary driver of chronic liver morbidity and mortality, and overwhelmingly towards conditions rooted in metabolic dysfunction (Feng et al., 2025; Gines et al., 2025). This shift is driven by the parallel global epidemics of type 2 diabetes mellitus (T2DM) and obesity, which fuel the rise of metabolic dysfunction-associated steatotic liver disease (MASLD) (Danpanichkul et al., 2025; Huang et al., 2025). Now recognized as the most common chronic liver condition globally, MASLD creates a niche for the development of advanced liver pathology, including cirrhosis and hepatocellular carcinoma (HCC) (Huang et al., 2025; Gao et al., 2025). MASLD is estimated to affect over 30% of the worldwide population, with prevalence rates exceeding 70% in individuals with T2DM, underscoring the severity of this metabolic-hepatic nexus (Garg and Zein, 2025).

This metabolic crisis is further complicated by alcohol-associated liver disease (ALD), an aggressive pathology resulting from excessive alcohol consumption. While ALD and MASLD were historically viewed as distinct entities, they share similar histology and some pathological mechanisms, most notably altered metabolic homeostasis in the liver and adipose tissue. More critically, the disease spectrum now includes metabolic dysfunction and alcohol-associated liver disease (MetALD), a recently classified subcategory (Kalligeros et al., 2024; Ho et al., 2025). In MetALD, the co-existence of metabolic risk factors (like obesity and T2DM) and moderate-to-high alcohol consumption creates a synergistic “double-hit” injury that accelerates disease progression and HCC risk significantly more than either factor alone. Collectively, the rising prevalence of MASLD, ALD and MetALD has made these conditions the fastest-growing cause of HCC and among the leading reason for liver transplantation worldwide (Ochoa-Allemant et al., 2025; Harris, 2025).

Furthermore, while chronic hepatitis B virus (HBV) and hepatitis C virus (HCV) infections remain established risk factors for HCC, they also induce changes in lipid and glucose homeostasis. This suggests that even viral infections rely on a shared pathophysiological link through altered metabolism and the induction of SLD to drive carcinogenesis (Wang and Zhang, 2023; Wang J. et al., 2024; Nakamura et al., 2025). Given the central role of altered metabolism across virtually all major HCC etiologies, the development of potent pharmacotherapies that target metabolic dysfunction is an urgent priority.

Glucagon-like peptide-1 receptor agonists (GLP-1 RAs) have emerged as a promising class of medications to address this underlying metabolic dysregulation that drives SLD. Initially developed for glycemic control in T2DM, GLP-1 RAs act as incretin mimetics by stimulating glucose-dependent insulin secretion and inhibiting glucagon release (Brunton and Wysham, 2020; He X. et al., 2024). Beyond their effects on insulin secretion, accumulating evidence demonstrates that GLP-1 RAs also enhance insulin sensitivity and reduce systemic insulin resistance. More recent studies further highlight that these agents exert both central and peripheral actions, conferring a powerful dual mechanism of action. Centrally, GLP-1 RAs modulate reward pathways to reduce cravings for both high-calorie foods and alcohol (Farokhnia et al., 2025; Jerlhag, 2025; Hendershot et al., 2025), key behavioral drivers of SLD. Peripherally, they improve insulin sensitivity, reduce hepatic lipotoxicity, and attenuate systemic and hepatic inflammation (Jones and Brierley, 2025; Skibicka, 2013). Clinical studies in patients with MASLD have shown that GLP-1 RA therapy is associated with clinically meaningful reductions in progression to metabolic dysfunction-associated steatohepatitis (MASH). Complementary preclinical studies in animal models demonstrate that GLP-1 RAs reduce alcohol craving and alcohol consumption, suggesting potential therapeutic benefit in alcohol use disorder (AUD). These central effects, together with their ability to ameliorate metabolic dysregulation observed in ALD, a condition for which the only established therapeutic options currently remain sustained abstinence or liver transplantation. This review aims to dissect the epidemiological and mechanistic links between altered metabolism and HCC across MASLD, ALD, and viral etiologies, summarize the current therapeutic landscape, and critically evaluate the emerging role of GLP-1 RAs as a strategic intervention for prevention and clinical management across this complex disease spectrum.

## 2 Global epidemiology and SLD progression to HCC

HCC is a significant global health threat, ranking as the fourth most common cancer and the second leading cause of cancer-related mortality worldwide (Bray et al., 2024). The global rise in HCC incidence is strongly and disproportionately correlated with the epidemic rise of MASLD (Ma et al., 2024; Shi et al., 2024). Metabolic dysfunction-associated steatohepatitis (MASH), the progressive form of MASLD characterized by inflammation and hepatocyte injury, is expected to overtake chronic hepatitis C as the most common indication for liver transplantation (Goldberg et al., 2017; Younossi et al., 2021). Data from the last 2 decades reveal a dramatic increase in HCC attributable to MASH (Karin and Kim, 2025). Alcohol consumption also remains an intractable risk factor, with heavy, long-term use contributing significantly to HCC and accounting for 30% of HCC cases and HCC-specific deaths (Patel and Mueller, 2025). This risk is further compounded in MetALD patients, where the coexisting metabolic syndrome and alcohol consumption establish a uniquely high-risk population driven by synergistic insults (Ochoa-Allemant et al., 2025; Harris, 2025). Regardless of the initial trigger (metabolic stress, alcohol, or virus), the disease typically progresses through a multi-step process: simple steatosis to steatohepatitis (MASH/severe ALD), to cirrhosis and HCC. Intervening at the steatosis or steatohepatitis stage is critical for effective prevention (Jee et al., 2025).

## 3 Spectrum of SLD and mechanisms of progression to HCC

HCC predominantly arises in the setting of chronic liver injury, but the specific insults (metabolic excess, alcohol, or viral infection) converge upon a common pathogenic pathway: dysregulated hepatic metabolism. This dysregulated metabolism creates an environment characterized by persistent oxidative stress, chronic inflammation, and altered energy signaling, which collectively drive fibrosis and cellular transformation (Ding et al., 2025; Galasso et al., 2024; Ramaite and Nkadimeng, 2025). Understanding the specific metabolic alterations within each major etiology is key to target the disease with a compound, which can help metabolic homeostasis, such as GLP-1 R analogs (Wang and Lu, 2025; Kanwal et al., 2024; Havranek et al., 2025).

### 3.1 Type 2 diabetes mellitus (T2DM) and metabolic dysfunction-associated steatotic liver disease (MASLD)

The global epidemic of T2DM is inextricably linked to the rising burden of MASLD and HCC. Diabetes affecting over 830 million people, which accounts for 14% of adults 18 years or older worldwide (Danpanichkul et al., 2025). Alarming projections indicate that its prevalence will increase by 25% in 2030 and 50% in 2045 (He KJ. et al., 2024). As the dominant form of the disease, T2DM accounts for over 95% of diabetic patients and is largely attributable to the obesity pandemic (Cusi, 2019; Cusi et al., 2025; Miller et al., 2025). Further, T2DM remains one of the most significant risk factors not only for the development of MASLD but also for the progression of liver fibrosis and the occurrence of liver‐related complications, including HCC (Hosseinkhan et al., 2025; Leith et al., 2024; Riley et al., 2024).

In the context of MASLD and T2DM, altered metabolism is the primary etiology. The root cause is systemic insulin resistance, typically driven by high calorie intake and positive energy balance (excess calories). This chronic caloric excess eventually overwhelms the storage capacity of visceral adipose tissue, leading to adipose tissue failure. Adipose tissue failure forces the spillover of excessive free fatty acids (FFA) into the systemic circulation, causing ectopic fat deposition in non-adipose organs, including the skeletal muscle and, critically, the liver (Dua et al., 2025; Stefan et al., 2025; Tantu et al., 2025). These high circulating FFAs, alongside inflammatory adipokines released by dysfunctional fat tissue, impair insulin signaling and develop systemic insulin resistance. This insulin resistance affects the liver directly, promoting increased *de novo* lipogenesis and endogenous glucose production, thereby driving both hepatic steatosis and hyperglycemia.

The transition from simple steatosis to advanced liver disease is governed by a multi-parallel hit model against this backdrop of established insulin resistance. Hepatic lipotoxicity creates a pro-inflammatory environment that makes the liver highly susceptible to simultaneous insults, including mitochondrial dysfunction (generating oxidative stress), endoplasmic reticulum stress (Miller et al., 2025; Tantu et al., 2025; Li et al., 2024) and the activation of innate immune cells (kupffer cells) by signals from inflammatory adipokines and endotoxins from the gut (due to gut dysbiosis). These parallel hits drive inflammation, hepatocellular ballooning, and subsequent fibrosis, forming the critical juncture that determines the risk of progression to cirrhosis and HCC.

Current management strategies include foundational interventions like calorie restriction and lifestyle modification, which are shown to improve MASLD treatment outcomes by addressing the root metabolic cause. Furthermore, hypoglycemic agents such as pioglitazone and empagliflozin exhibit beneficial effects on liver tissue outcomes, such as reducing liver fat content and improving liver fibrosis (Jee et al., 2025; Miller et al., 2025; Armandi and Bugianesi, 2024; Isaacs et al., 2025; Pieralice et al., 2025), demonstrating that therapeutic targeting of the metabolic axis is effective.

### 3.2 Alcohol-associated liver disease (ALD)

Excessive alcohol use is a serious health concern globally, and after the brain, the liver sustains the greatest damage. ALD is a major health problem, accounting for 30% of HCC cases (Choi and Kim, 2025). Ninety percent of individuals with excessive alcohol use develop steatosis, the earliest and most common response of the liver to excessive ethanol consumption. Compared to non-drinkers, heavy alcohol consumption has been associated with an 87% increased HCC risk (Chang et al., 2024; Pan et al., 2025).

Central among the many mechanisms proposed to play a role in the development of ALD are impaired metabolic homeostasis and organ crosstalk. Studies have shown that impaired levels of insulin and an increase in insulin resistance lead to increased adipose lipolysis and FFA release into the circulation and their enhanced hepatic uptake, subsequently causing fat accumulation in the liver (Zhong et al., 2012; Wei et al., 2013; Kang et al., 2007; Rasineni et al., 2019a; Boden et al., 2005; Rasineni et al., 2019b). In addition to increased uptake of adipose-derived FFA, several factors contribute to hepatic fat deposition: I) Increased fatty acid synthesis from ethanol-induced metabolism (increased NADH from ethanol metabolism induces fatty acid synthesis); II) Impaired fat transport out of the liver via reduced very-low-density lipoprotein (VLDL) secretion; III) Decreased fatty acid oxidation (Rasineni and Casey, 2012; Osna et al., 2022; Yan et al., 2023). The resulting accumulation of fat in hepatocytes makes the liver susceptible to inflammatory mediators and/or toxic agents, leading to progressive injury, which progresses to alcoholic steatohepatitis (ASH), fibrosis, cirrhosis, and cancer (Li et al., 2024; Mackowiak et al., 2024). The only effective therapeutic strategies currently available for ALD are alcohol abstinence or liver transplantation (Addolorato et al., 2013; Kharbanda et al., 2022; Leko and Leggio, 2024; Adekunle et al., 2023). Any molecule with dual-pronged effects at the central and peripheral organs controlling alcohol craving and alcohol-associated metabolic dysregulation could be a promising therapeutic target to treat alcohol use disorder (AUD) and ALD.

### 3.3 Metabolic dysfunction and alcohol-associated liver disease (MetALD)

The newly recognized subcategory, MetALD, highlights the potent synergy between toxic and metabolic insults. In this recently classified subcategory of liver disease, the co-existence of diet-induced obesity (a metabolic factor) and moderate to high amounts of alcohol consumption creates a double-hit injury. This combination synergistically increases disease progression and HCC risk significantly compared to obesity or alcohol alone (Gratacos-Gines et al., 2025; Marek and Malhi, 2024; Babuta et al., 2024; Schonfeld et al., 2021).

While the individual pathologies of MASLD and ALD are well-established, the specific pathophysiological mechanisms for MetALD development remain under active investigation and are currently considered hypothetical models that require further clinical validation. However, it is proposed that ethanol metabolism directly impairs the hepatic redox state by increasing the NADH/NAD^+^ ratio. This increased NADH promotes *de novo* lipogenesis while simultaneously decreasing fatty acid oxidation (Rasineni and Casey, 2012). Beyond the liver, alcohol administration disturbs lipid homeostasis within adipose tissue, triggering a pathological flux of FFA from peripheral fat depots to the liver (Zhong et al., 2012; Wei et al., 2013; Kang et al., 2007; Rasineni et al., 2019a; Boden et al., 2005; Rasineni et al., 2019b). This alcohol-induced steatosis is further compounded by the systemic insulin resistance from obesity, which independently drives an influx of FFA to the liver. Furthermore, the induction of CYP2E1 during chronic alcohol consumption generates massive amounts of ROS, which likely accelerates mitochondrial dysfunction and ER stress, and inflammatory signaling.

This detrimental progression of liver diseases is often driven by a behavioral cycle involving an increased desire, cravings, and high-calorie food intake during and after alcohol drinking episodes and *vice versa*. Notably, both MASLD and ALD exhibit similar histology and share some pathological mechanisms, reinforcing the idea that altered metabolic homeostasis in the liver and adipose tissue is the final common pathway driving liver injury (Kalligeros et al.; Ho et al., 2025; Marek and Malhi, 2024; Acierno et al., 2025; Leal-Lassalle et al., 2025). However, further studies are warranted to fully delineate the distinct pathophysiology of MetALD development, which is driven by both alcohol and metabolic dysfunction.

### 3.4 Chronic viral hepatitis (HBV and HCV)

While HBV and HCV are primarily known as infectious diseases, their persistent presence induces profound metabolic reprogramming specifically within the host liver cells (hepatocytes), which contributes significantly to the risk of advanced liver disease and HCC (Wang and Zhang, 2023; Suhail et al., 2022; R et al., 2022). This viral-induced dysmetabolism creates a powerful synergy with the underlying drivers of SLD (Schinzari et al., 2015).

HCV infection is strongly associated with systemic insulin resistance and T2DM (El-Zayadi and Anis, 2012). Critically, the virus achieves this by interfering with crucial host signaling pathways within the liver. Specifically, the HCV core protein is known to induce the degradation of insulin receptor substrate (IRS-1 and IRS-2) components, which are vital for proper insulin signaling in hepatocytes (Bose et al., 2012). This interference promotes IR at the cellular level, intensifying the burden of hyperinsulinemia and hyperglycemia on the liver (Kawaguchi et al., 2004; Pazienza et al., 2007).

Furthermore, HCV directly alters hepatic lipid metabolism in a way that mimics and exacerbates hepatic steatosis. The virus promotes the formation and accumulation of lipid droplets (LDs) within hepatocytes, a process essential for its own life cycle, including viral replication and assembly (Kim et al., 2007; Perlemuter et al., 2002). The resulting high levels of intrahepatic fat deposition, coupled with the persistent IR, accelerate the chronic inflammatory and fibrotic processes characteristic of advanced liver disease (Salmon et al., 2012).

HBV, a DNA virus, has also been shown to modulate host metabolic pathways to support its life cycle, with primary effects observed in the liver. Viral proteins, particularly the hepatitis B virus X protein (HBx), act as an important positive regulator for gluconeogenesis (Shin et al., 2011). Further, HBx interacts with cellular machinery to alter lipid homeostasis in the hepatocyte (Yang et al., 2008). HBx can influence the activity of metabolic transcription factors, such as sterol regulatory element-binding proteins (SREBPs), which are key regulators of *de novo* lipogenesis (Hajjou et al., 2005). This modulation leads to observable steatosis in the liver of some chronic carriers.

Thus, for viral etiologies, the virus acts as the initial trigger, but the resulting metabolic dysregulation and chronic inflammatory state which overlaps significantly with the pathophysiology of MASLD are focused on and ultimately destroy the liver’s tissue architecture, creating the sustained carcinogenic environment (Wang and Zhang, 2023; Suhail et al., 2022; R et al., 2022; Schinzari et al., 2015). Critically, even after successful viral eradication (e.g., sustained virologic response in HCV), residual metabolic damage within the liver can persist, leaving patients with a continued, metabolically-driven risk for HCC (Przybyszewski and Chung, 2023).

## 4 Current therapeutic landscape and unmet need

While resmetirom (a THR-β agonist) has recently gained FDA approval for the treatment of MASH (in patients with fibrosis F2-F3), marking the first-ever pharmacological agent for this condition (Liu et al., 2025), there remains no single FDA-approved drug that effectively prevents or reverses the progression of advanced SLD to HCC. The therapeutic approach for HCC remains focused on curative treatments for early stages (resection, ablation, transplantation) or palliative systemic therapies for advanced disease (tyrosine kinase inhibitors [TKIs] and VEGF inhibitors) and immunotherapies (i.e., programmed death 1 [PD-1]/programmed cell death-ligand 1 [PD-L1] inhibitors and anti-cytotoxic T-lymphocyte-associated protein 4 [CTLA-4] antibodies) (Sadagopan et al., 2024; Gord et al., 2024).

Management of the underlying SLDs typically revolves around etiology-specific interventions: lifestyle modifications (diet, exercise, and weight loss) are the cornerstone for MASLD/MASH; complete alcohol cessation (abstinence) is critical for ALD/MetALD; and antivirals are used to suppress viral load in chronic hepatitis (Hailemichael et al., 2024; Sengupta and Mellinger, 2024). However, while lifestyle modification (such as calorie restriction and alcohol abstinence) represents the therapeutic gold standard for MASLD and ALD, achieving and, more importantly, sustaining these intensive behavioral changes over the long term is a significant clinical challenge (Chen et al., 2024; Rajewski et al., 2025). This difficulty in maintaining persistent intervention means that the underlying metabolic dysfunction often continues as the primary driver of advanced liver disease, even in patients who have achieved viral cure or who struggle with these rigorous modifications. This gap defines a critical therapeutic void. There is an urgent need for a pharmacological agent that can specifically and safely correct the shared metabolic pathology, ideally by acting at both central (e.g., controlling appetite and cravings) and peripheral (e.g., improving insulin sensitivity, reducing hepatic fat and inflammation) levels to support and enhance existing behavioral strategies, thereby lowering the risk of advanced liver disease development across metabolic and alcoholic etiologies.

## 5 GLP-1 receptor agonists: a multi-functional therapeutic targeting metabolic dysfunction

**FIGURE 1:**
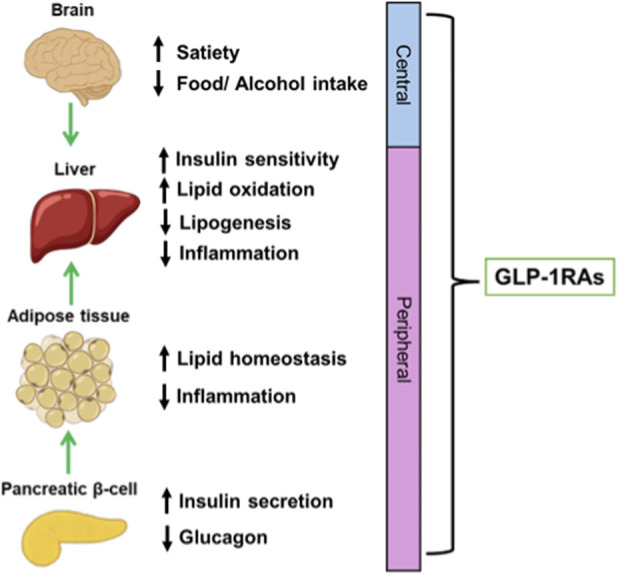
Pleiotropic Central and Peripheral Effects of GLP-1 Receptor Agonists (GLP-1RAs). This illustration depicts the integrated mechanisms of GLP-1RAs, highlighting their central modulation of satiety pathways, which suppresses both caloric and alcohol intake, alongside their extensive peripheral system effects. Key systemic actions include enhancing glucose-dependent insulin secretion, restoring insulin sensitivity, and improving systemic metabolic homeostasis.

Glucagon-like peptide-1 (GLP-1), a multifaceted hormone expressed primarily in the intestines and pancreas, mediates physiological and behavioral properties through its receptor, GLP-1 receptors (GLP-1R). The GLP-1R is widely expressed in both central and peripheral tissues, including the brain, heart, and kidney. Importantly, several studies have reported GLP-1R expression on both hepatocytes (liver cells) and adipocytes (fat cells) (Gupta et al., 2010; Zhang et al., 2025; Mahalingam et al., 2023; Vendrell et al., 2011; Muller et al., 2019). However, this remains an area of active debate within the field; while some studies report direct hepatocyte GLP-1R signaling (Shantaram et al., 2025; Hu et al., 2025; Sharma et al., 2011; Gupta et al., 2012; Ding et al., 2006; Wang et al., 2015), others suggest that many hepatic benefits may be indirect, mediated via weight loss, improved insulin sensitivity, and adipose-liver crosstalk (da Silva Lima et al., 2024). Regardless of the specific signaling site, the therapeutic potential of GLP-1 remains significant. Given the rapid degradation of the natural GLP-1 hormone (with a half-life of only ∼2 min), with therapeutic potential motivated the development of long-acting GLP-1 receptor agonists (GLP-1 RAs). These synthetic analogs possess significantly long half-life and are now used clinically. Originally developed as glucose-lowering agents for T2DM (Brunton and Wysham, 2020; He X. et al., 2024), GLP-1 RAs function as incretin mimetics, stimulating insulin release by pancreatic β-cells in a glucose-dependent manner while suppressing glucagon secretion from α-cells (Figure 1).

The mechanism of GLP-1 RAs, however, is now understood to extend far beyond glucose control, offering powerful dual central and peripheral action. Centrally, they manage appetite and cravings by modulating the reward circuitry in the brain, while peripherally, they correct systemic metabolism and reduce hepatic fat accumulation (Jones and Brierley, 2025; Skibicka, 2013; Shantaram et al., 2025; Weghuber et al., 2022; Cusi et al., 2018). This critical dual action is reinforced by data showing that GLP-1R agonists reduce the intake of both high-calorie food and alcohol, which are key drivers of SLDs (Farokhnia et al., 2025; Jerlhag, 2025; Hendershot et al., 2025; Weghuber et al., 2022; Cusi et al., 2018). This multi-functional activity positions GLP-1Rs as a class of medications that target the fundamental metabolic dysregulation driving SLD and the progression to advanced liver injury, thus establishing their role as a leading therapeutic strategy for managing the risk of advanced liver disease and HCC.

Importantly, this mechanistic framework is increasingly supported by large-scale clinical outcome data. Studies involving large patient cohorts demonstrate that GLP-1RA use is associated with a reduced all-cause mortality and decreased progression to decompensated cirrhosis among patients with MASLD/MASH. Furthermore, non-users of GLP-1RAs exhibited more than a twofold higher risk of all-cause mortality compared with GLP-1RA users (Kanwal et al., 2024; Havranek et al., 2025).

### 5.1 Systemic and hepatic efficacy: Correcting metabolic derangements and steatosis

As depicted in Figure 1, GLP-1 RAs are highly effective at correcting the core metabolic derangements seen in MASLD/T2DM. Their systemic efficacy is initiated by achieving significant and sustained weight loss and improved insulin sensitivity. This is accomplished centrally by reducing appetite and peripherally by delaying gastric emptying and enhancing glucose-dependent insulin secretion. These potent systemic actions lead to a reduction in visceral adiposity and dramatically improve systemic metabolic control (Weghuber et al., 2022; Cusi et al., 2018; Cai et al., 2025; Mocciaro et al., 2025).

These systemic improvements translate directly to the liver, where GLP-1 RAs provide potent hepatic benefits that extend beyond simple weight loss (Summary of studies evaluating the role of GLP-1RAs in SLDs presented in Table 1). Mechanistically, they directly promote hepatic glucose metabolism and reduce fat accumulation. They reduce *de novo* lipogenesis by inhibiting transcription factor sterol regulatory element-binding protein 1c (SREBP-1c), which is a master regulator of fatty acid synthesis (Shantaram et al., 2025; Bu et al., 2024). Concurrently, they promote fatty acid β-oxidation by increasing peroxisome proliferator-activated receptor alpha (PPARα), a transcriptional factor regulating genes involved in mitochondrial and peroxisomal fatty acid catabolism (Ding et al., 2006). This crucial dual action of reducing synthesis and increasing catabolism effectively decreases intrahepatic triglyceride content. Furthermore, by improving overall insulin sensitivity, GLP-1RAs decrease the toxic influx of free fatty acids from adipose tissue to the liver, thereby mitigating hepatic lipotoxicity (Wang et al., 2017). Beyond lipid metabolism, the GLP-1 RAs also shown critical anti-inflammatory and antioxidant effects, collectively positioning these GLP-1 RAs as a leading pharmacotherapy for MASLD (Chen et al., 2025; Pasta et al., 2025; Wang L. et al., 2024; Wei et al., 2025). Clinically, this potent effect is demonstrated by agents like semaglutide, which, in phase 2 trials, achieved MASH resolution without worsening fibrosis in a significant percentage of patients (Armstrong et al., 2016; Newsome et al., 2021; Petta et al., 2025; Sanyal et al., 2025; Lawitz et al., 2024; Jara et al., 2025).

**TABLE 1:** Therapeutic actions of GLP-1 receptor agonists in liver disease: summary of experimental and clinical findings.

GLP-1RAs	Animal model/clinical study	Biological actions	Level of evidence	References
Exenatide (Ex-4)	Clinical trial	Exenatide decreased alcohol intake in overweight alcohol use disorder (AUD) patients	Clinical	Klausen et al. (2022)
Dulaglutide	Clinical study	Dulaglutide treatment decreased alcohol intake in smoking AUD patients	Clinical	Probst et al. (2023)
Semaglutide and tirzepatide	Clinical study	Semaglutide lowered binge drinking, alcohol use disorder identification test (AUDIT) scores, and self-reported alcohol intake in overweight individuals with high alcohol intake	Clinical	(Quddos et al., 2023)
Exendin-4	Lieber-DeCarli ethanol-diet fed rat	Exendin-4 reduces hepatic steatosis in Lieber-DeCarli ethanol-diet fed animals by improving insulin signaling and fat metabolism	Clinical	Mahalingam et al. (2023)
GLP-1RAs	Clinical study	GLP-1R agonist treatment reduces the desire to consume alcohol, interest in alcohol, and alcohol consumption in patients treated for obesity or type 2 diabetes	Clinical	Bremmer and Hendershot (2024)
GLP-1RAs	Clinical and experimental animal study	GLP-1RAs reduce alcohol consumption	Clinical	Farokhnia et al. (2025)
Semaglutide	Clinical trial	Low-dose semaglutide over 9 weeks of treatment can reduce alcohol craving and some drinking outcomes	Clinical	Hendershot et al. (2025)
Liraglutide	HepG2 cells	HepG2 cells decreased proliferation after liraglutide treatment without altering oxidative stress levels. Liraglutide was able to induce autophagy and senescence through the increase of TGF-β1, which possibly explains the growth decrease	*In vitro*	Krause et al. (2017)
Exenatide (Ex-4)	Obese DEN-treated mice	Ex-4 significantly improved obesity-induced hyperglycemia and hyperlipidemia and reduced HCC multiplicity in obese DEN-treated mice, in which suppressed proliferation and induced apoptosis were confined to tumor cells	*In vivo*	Zhou et al. (2017)
Exenatide (Ex-4)	HepG2 cells	Exenatide has a potent anti-proliferative activity via mTOR modulation using HepG2 cells	*In vitro*	Krause et al. (2019)
Liraglutide	Mouse	Liraglutide prevented the progression of hepatocellular carcinoma in a mouse model of Nonalcoholic Steatohepatitis	*In vivo*	Kojima et al. (2020)
Liraglutide	Mouse	Liraglutide activates natural killer cell-mediated antitumor responses by inhibiting IL-6/STAT3 signaling in hepatocellular carcinoma	*In vivo*	Lu et al. (2021)
Semaglutide	GAN-DIO‐NASH mice	Semaglutide prevents NASH-driven HCC progression in a metabolic translational preclinical model	*In vivo*	Mollerhoj et al. (2022)
Liraglutide	HFD mice	Liraglutide ameliorates hepatic steatosis via retinoic acid receptor-related orphan receptor α-mediated autophagy pathway	*In vivo*	Yu et al. (2023)
Semaglutide	GAN diet-induced obese	Semaglutide improves both NASH and tumor burden in GAN DIO-NASH-HCC mice, highlighting the suitability of this preclinical model for profiling novel drug therapies targeting NASH-HCC.	*In vivo*	Hansen et al. (2023)
Semaglutide	HFD-induced obese mice	Semaglutide treatment decelerates the progression of liver cancer by inducing the expression of ITGAV, LAMC1, FABP5, and LPL in the adipose tissue of obese mice	*In vivo*	Liu et al. (2024)
GLP-1R agonists	Meta-analysis	GLP-1 RAs provide protective benefits against HCC in T2DM patients compared to insulin or no GLP-1 RAs, but not significantly over other antidiabetic medications	Meta-analysis	Shabil et al. (2024)
GLP-1R agonists	Meta-analysis	GLP-1RAs were associated with significant risk reductions in long-term adverse liver outcomes, including hepatic decompensation, portal hypertension, HCC, and LT, in MASLD cirrhosis patients with type 2 diabetes	Meta-analysis	Elsaid et al. (2024)
Semaglutide	MASH or Advanced liver fibrosis	Semaglutide at a dose of 2.4 mg improved liver histologic results in patients with MASH or advanced liver fibrosis	Clinical	Sanyal et al. (2025)
GLP-1R agonists	Meta-analysis	Meta-analysis provides evidence for the effectiveness of GLP-1 receptor agonists in managing MAFLD, with dual GLP-1/GIP agonists demonstrating superior hepatic benefits	Meta-analysis	Tamilwanan et al. (2025)

**Abbreviations used in the table:** HepG2: human liver cancer cell line; TGF-β1: transforming growth factor beta 1; HCC: hepatocellular carcinoma; DEN: diethylnitrosamine; mTOR: mechanistic target of rapamycin; IL-6: interleukin-6; STAT3: signal transducer and activator of transcription 3; NASH: nonalcoholic steatohepatitis; GAN DIO: gubra amylin NASH, diet-induced obese; LAMC1: laminin subunit gamma 1; FABP5:fatty acid binding protein 5; LPL: lipoprotein lipase; GLP-1, RA: glucagon-like peptide-1, receptor agonist; LT: liver transplant; MASLD: metabolic dysfunction-associated steatotic liver disease; MASH: metabolic dysfunction-associated steatohepatitis; MAFLD: metabolic associated fatty liver disease; GIP: glucose-dependent insulinotropic polypeptide; AUD: alcohol use disorder; AUDIT: alcohol use disorders identification test.

Furthermore, liraglutide and semaglutide have both demonstrated anti-fibrotic effects and their use in preclinical models prevents NASH-driven HCC progression (Sanyal et al., 2025; Mantovani et al., 2021). Beyond MASLD, emerging preclinical evidence highlights the therapeutic potential of GLP-1 RAs in ALD and MetALD (Singal and Leggio, 2025). Specifically, our published data confirms that exendin-4, significantly improves ALD pathology by reducing hepatic steatosis, inflammation, and oxidative stress in experimental rats (Mahalingam et al., 2023). In addition to our study, a recent clinical cohort study indicated that GLP-1 RA use was associated with significantly fewer liver-related outcomes. This includes reduced rates of acute decompensated liver failure and alcoholic hepatitis, although, notably, no significant difference was observed in the progression to cirrhosis within the study period (Wickramarachchi et al., 2025).

The central action of GLP-1 receptor agonists is supported by evidence showing that exenatide and semaglutide reduce the intake of high-calorie foods and alcohol, as well as relapse-like drinking in rodent models. These critical effects are mediated through GLP-1R signaling within the mesolimbic reward system, which directly targets key drivers of AUD, ALD, and MetALD. Preclinical and clinical studies have demonstrated that GLP-1RAs reduce both alcohol craving and intake, confirming the direct neurobiological pathway targeted by this drug class (Farokhnia et al., 2025; Jerlhag, 2025; Hendershot et al., 2025; Chuong et al., 2023; Klausen et al., 2022; Patel et al., 2025). This universality even extends to viral hepatitis, as liraglutide has been shown *in vitro* to directly inhibit HCV replication through an AMPK-dependent mechanism (Lee et al., 2019), reinforcing the drug class’s broad impact on metabolic health critical for diverse liver pathologies. This combined systemic, direct hepatic, anti-addictive, and even anti-viral action establishes GLP-1RAs as a powerful, novel approach to reduce the cumulative toxic insults driving SLD progression across multiple etiologies.

### 5.2 Anti-inflammatory and direct tumor-suppressive signaling

GLP-1 receptor agonists exert direct modulatory effects on some molecular pathways implicated in HCC initiation and progression, effects that may occur independently of reducing visceral adipose tissue and pro-inflammatory adipokine release from adipose tissue. Recognizing that chronic inflammation is a major driver of SLD progression and hepatocarcinogenesis, GLP-1 RAs demonstrate potent anti-inflammatory and tumor-suppressive actions (Que et al., 2019; Wan and Sun, 2019; Kojima et al., 2020; Zhao et al., 2014). The core tumor-suppressive mechanism involves activating AMP-activated protein kinase (AMPK), a key cellular energy sensor. AMPK activation subsequently inhibits the mTORC pathway (a major regulator of cell growth and proliferation) and promotes cell cycle arrest by stabilizing p53 and the p21/p27 inhibitors (Hardie, 2013; Hardie et al., 2012). This direct molecular targeting underpins their utility as specific anti-cancer agents.

Preclinical studies validate these direct anti-tumor effects across different agents: Liraglutide, for instance, demonstrates potent anti-cancer activity by suppressing HCC progression in mouse models, crucially exhibiting an anti-inflammatory effect by inhibiting the IL-6/STAT3 signaling pathway (Kojima et al., 2020; Lu et al., 2021). This action enhances antitumor activity via NK-mediated cytotoxicity, while liraglutide also induces autophagy and senescence in HepG2 cells via TGF-β modulation (Chen-Liaw et al., 2017; Krause et al., 2017). Similarly, exenatide-4 inhibits hepatocarcinogenesis independent of obesity and MASH resolution, acting through the cAMP-PKA-EGFR-STAT3 axis and mTOR modulation to suppress proliferation and reduce HCC multiplicity (Zhou et al., 2017). Furthermore, semaglutide prevents MASLD/MASH-driven HCC progression in metabolic preclinical models, partly by correcting adipokine imbalances (low adiponectin, high leptin) associated with insulin resistance, which removes a key chronic inflammatory and pro-oncogenic signal linked to the disease (Kojima et al., 2020).

It is important to note that most of the evidence supporting the anti-tumor efficacy of GLP-1 RAs is derived from preclinical studies, including *in vitro* studies and animal experiments demonstrating reduced proliferation of the cancer cells, enhanced apoptosis, and modulation of oncogenic signaling pathways (Arvanitakis et al., 2022). In humans, current data remain largely associative, based on observational and epidemiological studies reporting lower HCC incidence among GLP-1 RA users compared to placebo. However, there are no randomized controlled trials with HCC incidence as a primary endpoint, and the observed associations may be influenced by confounding factors such as weight reduction, improved glycemic control, and concomitant use of statins or other hepatoprotective agents. These limitations underscore the need for prospective, well-designed clinical trials to establish causality and clarify the direct role of GLP-1 RAs in HCC prevention.

These systemic and direct molecular effects, particularly the potent anti-inflammatory actions and metabolic correction, are highly relevant to mitigating the progression of both ALD and MetALD. By resolving the underlying steatosis and reducing the chronic inflammatory burden, GLP-1 RAs reinforce their potential as powerful anticancer agents across the full spectrum of SLD. However, further preclinical studies, especially those focusing on ALD and MetALD models, and prospective clinical trials are necessary to fully translate these promising anticancer benefits to human patients.

### 5.3 Tolerability and safety profile of GLP-1 receptor agonists

GLP-1 receptor agonists are generally well tolerated; however, gastrointestinal adverse effects such as nausea, vomiting, and diarrhea are the most frequently reported, particularly during the initial treatment phase (Wilding et al., 2021; Wharton et al., 2022; Jastreboff et al., 2022; Ghusn and Hurtado, 2024). These symptoms are typically transient but may lead to treatment discontinuation in approximately 5%–10% of patients. Less frequent but clinically relevant risks include pancreatitis and gallbladder disease, such as cholelithiasis and cholecystitis (Sodhi et al., 2023; He et al., 2022). Although a causal association with acute pancreatitis remains debated, observational studies suggest a higher incidence compared with placebo (Nieto et al., 2025; Storgaard et al., 2017; Pinto et al., 2019). GLP-1 RAs also delay gastric emptying, which contributes to satiety but may exacerbate symptoms in patients with pre-existing gastroparesis (He et al., 2022; Jalleh et al., 2024).

Additionally, weight loss with GLP-1 RAs is accompanied by reductions in lean mass, with skeletal muscle accounting for approximately 15%–40% of total weight loss (Pandey et al., 2024; Sargeant et al., 2019), raising significant concerns regarding sarcopenia, particularly in older or frail individuals with cirrhosis. Caution is advised in patients with a history of severe gastrointestinal disorders or pancreatitis. While these adverse effects do not typically outweigh the substantial metabolic benefits, they may limit use in specific patient populations, necessitating careful dose titration and clinical monitoring. Also, side effects for GLP-1RAs in liver disease patients often include gastrointestinal issues, which can be managed with lower initial doses.

## 6 Conclusion and future directions

**FIGURE 2:**
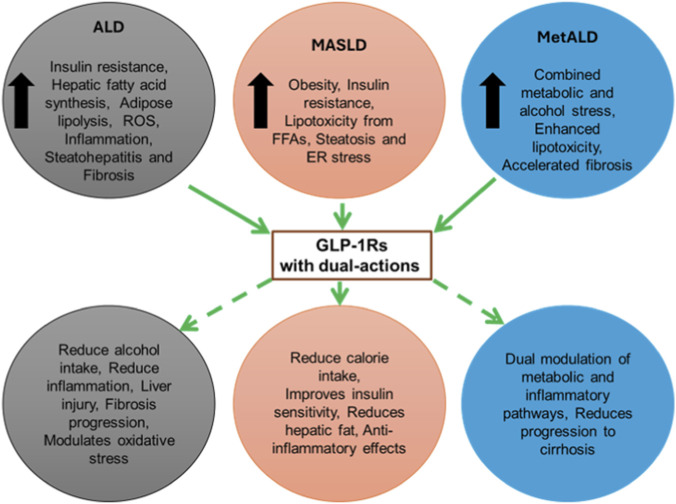
Mechanistic overview of GLP-1 Receptor agonists (GLP-1 RAs) in ALD, MASLD and MetALD. This schematic illustrates the proposed mechanisms by which GLP-1RAs can exert beneficial effects across the spectrum of steatotic liver diseases, including alcohol-associated liver disease (ALD), metabolic dysfunction-associated steatotic liver disease (MASLD), and metabolic dysfunction and alcohol-associated liver disease (MetALD).

The data strongly supports that SLD, is fueled by common pathways of altered metabolism and chronic inflammation, which are the central precipitating factors for HCC development across MASLD, ALD, and to some extent, chronic viral etiologies. Given this unifying pathophysiology, GLP-1 RAs, with their powerful ability to act both centrally (controlling appetite and cravings) and peripherally (correcting systemic metabolic dysfunction, resolving hepatic steatosis and inflammation), to exert direct anti-carcinogenic effects, represent a truly multi-functional therapeutic tool. Their ability to simultaneously address metabolic risk factors, inflammation, and cellular proliferation positions them as one of the most promising drug classes for interrupting SLD progression (Figure 2).

The encouraging efficacy of GLP-1 RAs in reversing MASH histology in clinical trials and reducing HCC risk in epidemiological studies provides a powerful mandate for further investigation. However, the next phase of translational research must now broaden its scope. Specifically, future work should prioritize conducting specific animal studies to validate the hypothesized anti-fibrotic and anti-cancer effects of GLP-1 RAs in ALD and MetALD models.

Crucially, more clinical studies are required to definitively establish the efficacy and safety of GLP-1 RAs for the treatment of ALD and MetALD. These trials must move beyond MASLD and include populations with significant alcohol exposure and mixed etiologies. Furthermore, research is critical to investigate the impact of GLP-1 RAs on HCC risk in patients with significant metabolic risk factors, including those who have achieved viral suppression from chronic viral hepatitis. Ultimately, the definitive role of GLP-1 RAs as an anticancer agent pivot on launching large-scale, randomized controlled trials focused explicitly on the primary prevention of HCC in high-risk patients with advanced SLD of all major etiologies.

## References

[B1] Acierno C. Barletta F. Caturano A. Nevola R. Sasso F. C. Adinolfi L. E. (2025). Alcohol consumption and liver metabolism in the era of MASLD: integrating nutritional and pathophysiological insights. Nutrients 17 (13), 2229. 10.3390/nu17132229 40647333 PMC12251479

[B2] Addolorato G. Mirijello A. Leggio L. Ferrulli A. Landolfi R. (2013). Management of alcohol dependence in patients with liver disease. CNS Drugs 27 (4), 287–299. 10.1007/s40263-013-0043-4 23456576 PMC4979989

[B3] Adekunle A. D. Adejumo A. Singal A. K. (2023). Therapeutic targets in alcohol-associated liver disease: progress and challenges. Ther. Adv. Gastroenterol. 16, 17562848231170946. 10.1177/17562848231170946 37187673 PMC10176580

[B4] Armandi A. Bugianesi E. (2024). Dietary and pharmacological treatment in patients with metabolic-dysfunction associated steatotic liver disease. Eur. J. Intern Med. 122, 20–27. 10.1016/j.ejim.2024.01.005 38262842

[B5] Armstrong M. J. Hull D. Guo K. Barton D. Hazlehurst J. M. Gathercole L. L. (2016). Glucagon-like peptide 1 decreases lipotoxicity in non-alcoholic steatohepatitis. J. Hepatol. 64 (2), 399–408. 10.1016/j.jhep.2015.08.038 26394161 PMC4713865

[B6] Arvanitakis K. Koufakis T. Kotsa K. Germanidis G. (2022). How far beyond diabetes can the benefits of glucagon-like Peptide-1 receptor agonists go? A review of the evidence on their effects on hepatocellular carcinoma. Cancers (Basel) 14 (19), 4651. 10.3390/cancers14194651 36230573 PMC9562923

[B7] Babuta M. Morel C. de Carvalho Ribeiro M. Datta A. A. Calenda C. Copeland C. (2024). A novel experimental model of MetALD in male mice recapitulates key features of severe alcohol-associated hepatitis. Hepatol. Commun. 8 (7), e0450. 10.1097/HC9.0000000000000450 38896082 PMC11186819

[B8] Boden G. She P. Mozzoli M. Cheung P. Gumireddy K. Reddy P. (2005). Free fatty acids produce insulin resistance and activate the proinflammatory nuclear factor-kappaB pathway in rat liver. Diabetes 54 (12), 3458–3465. 10.2337/diabetes.54.12.3458 16306362

[B9] Bose S. K. Shrivastava S. Meyer K. Ray R. B. Ray R. (2012). Hepatitis C virus activates the mTOR/S6K1 signaling pathway in inhibiting IRS-1 function for insulin resistance. J. Virol. 86 (11), 6315–6322. 10.1128/JVI.00050-12 22457523 PMC3372214

[B10] Bray F. Laversanne M. Sung H. Ferlay J. Siegel R. L. Soerjomataram I. (2024). Global cancer statistics 2022: GLOBOCAN estimates of incidence and mortality worldwide for 36 cancers in 185 countries. CA Cancer J. Clin. 74 (3), 229–263. 10.3322/caac.21834 38572751

[B11] Bremmer M. P. Hendershot C. S. (2024). Social media as pharmacovigilance: the potential for patient reports to inform clinical research on glucagon-like peptide 1 (GLP-1) receptor agonists for substance use disorders. J. Stud. Alcohol Drugs 85 (1), 5–11. 10.15288/jsad.23-00318 37917019 PMC10846600

[B12] Brunton S. A. Wysham C. H. (2020). GLP-1 receptor agonists in the treatment of type 2 diabetes: role and clinical experience to date. Postgrad. Med. 132 (Suppl. 2), 3–14. 10.1080/00325481.2020.1798099 32815454

[B13] Bu T. Sun Z. Pan Y. Deng X. Yuan G. (2024). Glucagon-like Peptide-1: new regulator in lipid metabolism. Diabetes Metab. J. 48 (3), 354–372. 10.4093/dmj.2023.0277 38650100 PMC11140404

[B14] Cai Y. Xie S. Xu L. Chen J. Cai J. (2025). Systemic evaluation of the effects of monomeric GLP-1R-based agonists on MASLD and its complications. Diabetol. Metab. Syndr. 17 (1), 300. 10.1186/s13098-025-01870-x 40731367 PMC12305972

[B15] Chang B. Tian H. Huang A. Zhai X. Wang Q. Han L. (2024). Prevalence and prediction of hepatocellular carcinoma in alcohol-associated liver disease: a retrospective study of 136 571 patients with chronic liver diseases. eGastroenterology 2 (1), e100036. 10.1136/egastro-2023-100036 39944749 PMC11731072

[B16] Chen M. J. Chen Y. Lin J. Q. Hu R. Liu D. Chen J. Y. (2024). Evidence summary of lifestyle interventions in adults with metabolic dysfunction-associated steatotic liver disease. Front. Nutr. 11, 1421386. 10.3389/fnut.2024.1421386 39834455 PMC11742927

[B17] Chen W. M. Ng H. J. Jao A. T. Wu S. Y. Soong R. S. (2025). GLP-1 receptor agonists and risk of hepatocellular carcinoma and all-cause mortality in patients with MASLD and type 2 diabetes: a propensity score-matched population-based cohort study. Diabetes Res. Clin. Pract. 227, 112407. 10.1016/j.diabres.2025.112407 40803507

[B18] Chen-Liaw A. Y. Hammel G. Gomez G. (2017). Inhibition of exendin-4-induced steatosis by protein kinase A in cultured HepG2 human hepatoma cells. Vitro Cell Dev. Biol. Anim. 53 (8), 721–727. 10.1007/s11626-017-0181-y 28707223

[B19] Choi J. Kim H.-s. (2025). Alcohol-associated liver disease and risk stratification for hepatocellular carcinoma: a comprehensive review. Curr. Hepatol. Rep. 24 (1), 13. 10.1007/s11901-025-00684-9

[B20] Chuong V. Farokhnia M. Khom S. Pince C. L. Elvig S. K. Vlkolinsky R. (2023). The glucagon-like peptide-1 (GLP-1) analogue semaglutide reduces alcohol drinking and modulates central GABA neurotransmission. JCI Insight 8 (12), e170671. 10.1172/jci.insight.170671 37192005 PMC10371247

[B21] Cusi K. (2019). Incretin-based therapies for the management of nonalcoholic fatty liver disease in patients with type 2 diabetes. Hepatology 69 (6), 2318–2322. 10.1002/hep.30670 31006135

[B22] Cusi K. Sattar N. Garcia-Perez L. E. Pavo I. Yu M. Robertson K. E. (2018). Dulaglutide decreases plasma aminotransferases in people with Type 2 diabetes in a pattern consistent with liver fat reduction: a post hoc analysis of the AWARD programme. Diabet. Med. 35 (10), 1434–1439. 10.1111/dme.13697 29869810

[B23] Cusi K. Abdelmalek M. F. Apovian C. M. Balapattabi K. Bannuru R. R. Barb D. (2025). Metabolic dysfunction-associated steatotic liver disease (MASLD) in people with diabetes: the need for screening and early intervention. A consensus report of the American diabetes association. Diabetes Care 48 (7), 1057–1082. 10.2337/dci24-0094 40434108

[B24] da Silva Lima N. Cabaleiro A. Novoa E. Riobello C. Knerr P. J. He Y. (2024). GLP-1 and GIP agonism has no direct actions in human hepatocytes or hepatic stellate cells. Cell Mol. Life Sci. 81 (1), 468. 10.1007/s00018-024-05507-6 39607493 PMC11604888

[B25] Danpanichkul P. Suparan K. Diaz L. A. Fallon M. B. Chen V. L. Namsathimaphorn K. (2025). The rising global burden of MASLD and other metabolic diseases (2000-2021). United Eur. Gastroenterol. J. 13 (7), 1141–1154. 10.1002/ueg2.70072 40605557 PMC12463718

[B26] Ding X. Saxena N. K. Lin S. Gupta N. A. Anania F. A. (2006). Exendin-4, a glucagon-like protein-1 (GLP-1) receptor agonist, reverses hepatic steatosis in ob/ob mice. Hepatology 43 (1), 173–181. 10.1002/hep.21006 16374859 PMC2925424

[B27] Ding Z. Wang L. Sun J. Zheng L. Tang Y. Tang H. (2025). Hepatocellular carcinoma: pathogenesis, molecular mechanisms, and treatment advances. Front. Oncol. 15, 1526206. 10.3389/fonc.2025.1526206 40265012 PMC12011620

[B28] Dua A. Kumari R. Singh M. Kumar R. Pradeep S. Ojesina A. I. (2025). Metabolic dysfunction-associated steatotic liver disease (MASLD): the interplay of gut microbiome, insulin resistance, and diabetes. Front. Med. (Lausanne) 12, 1618275. 10.3389/fmed.2025.1618275 40893881 PMC12392282

[B29] El-Zayadi A. R. Anis M. (2012). Hepatitis C virus induced insulin resistance impairs response to anti viral therapy. World J. Gastroenterol. 18 (3), 212–224. 10.3748/wjg.v18.i3.212 22294824 PMC3261538

[B30] Elsaid M. I. Li N. Firkins S. A. Rustgi V. K. Paskett E. D. Acharya C. (2024). Impacts of glucagon-like peptide-1 receptor agonists on the risk of adverse liver outcomes in patients with metabolic dysfunction-associated steatotic liver disease cirrhosis and type 2 diabetes. Aliment. Pharmacol. Ther. 59 (9), 1096–1110. 10.1111/apt.17925 38538967

[B31] Farokhnia M. Tazare J. Pince C. L. Bruns Nt Gray J. C. Lo Re V. 3rd (2025). Glucagon-like peptide-1 receptor agonists, but not dipeptidyl peptidase-4 inhibitors, reduce alcohol intake. J. Clin. Invest. 135 (9), e188314. 10.1172/JCI188314 40048376 PMC12043080

[B32] Feng G. Targher G. Byrne C. D. Yilmaz Y. Wai-Sun Wong V. Adithya Lesmana C. R. (2025). Global burden of metabolic dysfunction-associated steatotic liver disease, 2010 to 2021. JHEP Rep. 7 (3), 101271. 10.1016/j.jhepr.2024.101271 39980749 PMC11840544

[B33] Galasso L. Cerrito L. Maccauro V. Termite F. Mignini I. Esposto G. (2024). Inflammatory response in the pathogenesis and treatment of hepatocellular carcinoma: a double-edged weapon. Int. J. Mol. Sci. 25 (13), 7191. 10.3390/ijms25137191 39000296 PMC11241080

[B34] Gao B. Lu Y. Lai X. Xu X. Gou S. Yang Z. (2025). Metabolic reprogramming in hepatocellular carcinoma: mechanisms of immune evasion and therapeutic implications. Front. Immunol. 16, 1592837. 10.3389/fimmu.2025.1592837 40370433 PMC12075234

[B35] Garg S. Zein N. N. (2025). Emerging trends in MAFLD and MASH. Diabetes Technol. Ther. 27 (S1), S227–S237. 10.1089/dia.2025.8818.sg 40094495

[B36] Ghusn W. Hurtado M. D. (2024). Glucagon-like Receptor-1 agonists for obesity: weight loss outcomes, tolerability, side effects, and risks. Obes. Pillars 12, 100127. 10.1016/j.obpill.2024.100127 39286601 PMC11404059

[B37] Gines P. Serra-Burriel M. Kamath P. S. (2025). Metabolic dysfunction-associated steatotic liver disease-the new epidemic of chronic liver disease. JAMA Netw. Open 8 (6), e2516381. 10.1001/jamanetworkopen.2025.16381 40526389

[B38] Goldberg D. Ditah I. C. Saeian K. Lalehzari M. Aronsohn A. Gorospe E. C. (2017). Changes in the prevalence of hepatitis C virus infection, nonalcoholic steatohepatitis, and alcoholic liver disease among patients with cirrhosis or liver failure on the waitlist for liver transplantation. Gastroenterology 152 (5), 1090–9 e1. 10.1053/j.gastro.2017.01.003 28088461 PMC5367965

[B39] Gordan J. D. Kennedy E. B. Abou-Alfa G. K. Beal E. Finn R. S. Gade T. P. (2024). Systemic therapy for advanced hepatocellular carcinoma: ASCO guideline update. J. Clin. Oncol. 42 (15), 1830–1850. 10.1200/JCO.23.02745 38502889

[B40] Gratacos-Gines J. Arino S. Sancho-Bru P. Bataller R. Pose E. (2025). MetALD: clinical aspects, pathophysiology and treatment. JHEP Rep. 7 (2), 101250. 10.1016/j.jhepr.2024.101250 39897615 PMC11782861

[B41] Gupta N. A. Mells J. Dunham R. M. Grakoui A. Handy J. Saxena N. K. (2010). Glucagon-like peptide-1 receptor is present on human hepatocytes and has a direct role in decreasing hepatic steatosis *in vitro* by modulating elements of the insulin signaling pathway. Hepatology 51 (5), 1584–1592. 10.1002/hep.23569 20225248 PMC2862093

[B42] Gupta N. A. Kolachala V. L. Jiang R. Abramowsky C. Romero R. Fifadara N. (2012). The glucagon-like peptide-1 receptor agonist Exendin 4 has a protective role in ischemic injury of lean and steatotic liver by inhibiting cell death and stimulating lipolysis. Am. J. Pathol. 181 (5), 1693–1701. 10.1016/j.ajpath.2012.07.015 22960075 PMC5691335

[B43] Hailemichael D. Renata F. David H. Francisco I. Daniel C. Luis Antonio D. (2024). Prevention and control of risk factors in metabolic and alcohol-associated steatotic liver disease. Metabolism Target Organ Damage 4 (3), 25. 10.20517/mtod.2024.30

[B44] Hajjou M. Norel R. Carver R. Marion P. Cullen J. Rogler L. E. (2005). cDNA microarray analysis of HBV transgenic mouse liver identifies genes in lipid biosynthetic and growth control pathways affected by HBV. J. Med. Virol. 77 (1), 57–65. 10.1002/jmv.20427 16032730

[B45] Hansen H. H. Pors S. Andersen M. W. Vyberg M. Nohr-Meldgaard J. Nielsen M. H. (2023). Semaglutide reduces tumor burden in the GAN diet-induced obese and biopsy-confirmed mouse model of NASH-HCC with advanced fibrosis. Sci. Rep. 13 (1), 23056. 10.1038/s41598-023-50328-5 38155202 PMC10754821

[B46] Hardie D. G. (2013). AMPK: a target for drugs and natural products with effects on both diabetes and cancer. Diabetes 62 (7), 2164–2172. 10.2337/db13-0368 23801715 PMC3712072

[B47] Hardie D. G. Ross F. A. Hawley S. A. (2012). AMPK: a nutrient and energy sensor that maintains energy homeostasis. Nat. Rev. Mol. Cell Biol. 13 (4), 251–262. 10.1038/nrm3311 22436748 PMC5726489

[B48] Harris K. (2025). An unmet need in MetALD. Hepatology 81 (2), 385–386. 10.1097/HEP.0000000000001168 39817724

[B49] Havranek B. Loh R. Torre B. Redfield R. Halegoua-DeMarzio D. (2025). Glucagon-like peptide-1 receptor agonists improve metabolic dysfunction-associated steatotic liver disease outcomes. Sci. Rep. 15 (1), 4947. 10.1038/s41598-025-89408-z 39930071 PMC11811119

[B50] He L. Wang J. Ping F. Yang N. Huang J. Li Y. (2022). Association of glucagon-like Peptide-1 receptor agonist use with risk of gallbladder and biliary diseases: a systematic review and meta-analysis of randomized clinical trials. JAMA Intern Med. 182 (5), 513–519. 10.1001/jamainternmed.2022.0338 35344001 PMC8961394

[B51] He X. Zhao W. Li P. Zhang Y. Li G. Su H. (2024a). Research progress of GLP-1RAs in the treatment of type 2 diabetes mellitus. Front. Pharmacol. 15, 1483792. 10.3389/fphar.2024.1483792 39902077 PMC11788294

[B52] He K. J. Wang H. Xu J. Gong G. Liu X. Guan H. (2024b). Global burden of type 2 diabetes mellitus from 1990 to 2021, with projections of prevalence to 2044: a systematic analysis across SDI levels for the global burden of disease study 2021. Front. Endocrinol. (Lausanne) 15, 1501690. 10.3389/fendo.2024.1501690 39583961 PMC11581865

[B53] Hendershot C. S. Bremmer M. P. Paladino M. B. Kostantinis G. Gilmore T. A. Sullivan N. R. (2025). Once-weekly semaglutide in adults with alcohol use disorder: a randomized clinical trial. JAMA Psychiatry 82 (4), 395–405. 10.1001/jamapsychiatry.2024.4789 39937469 PMC11822619

[B54] Ho G. J. K. Tan F. X. N. Sasikumar N. A. Tham E. K. J. Ko D. Kim D. H. (2025). High global prevalence of steatotic liver disease and associated subtypes: a meta-analysis. Clin. Gastroenterol. Hepatol. 23 (13), 2423–2432. 10.1016/j.cgh.2025.02.006 40204206

[B55] Hosseinkhan N. Najafi L. Jahangiri S. Emami Z. Khamseh M. E. (2025). Statin use and risk of HCC in patients with MASLD and T2DM: an umbrella review and meta-analysis. BMC Cancer 25 (1), 875. 10.1186/s12885-025-14299-2 40369443 PMC12080120

[B56] Hu Q. Zhang L. Tao Y. Xie S. Wang A. Luo C. (2025). Semaglutide ameliorates hepatocyte steatosis in a cell Co-Culture system by downregulating the IRE1alpha-XBP1-C/EBPalpha signaling pathway in macrophages. Pharmacology 110 (1), 26–35. 10.1159/000540654 39089233

[B57] Huang M. Chen H. Wang H. Zhang Y. Li L. Lan Y. (2025). Global burden and risk factors of MASLD: trends from 1990 to 2021 and predictions to 2030. Intern Emerg. Med. 20 (4), 1013–1024. 10.1007/s11739-025-03895-6 40019669 PMC12130103

[B58] Isaacs S. D. Farrelly F. V. Brennan P. N. (2025). Role of anti-diabetic medications in the management of MASLD. Frontline Gastroenterol. 16 (3), 239–249. 10.1136/flgastro-2024-102856

[B59] Jalleh R. J. Plummer M. P. Marathe C. S. Umapathysivam M. M. Quast D. R. Rayner C. K. (2024). Clinical consequences of delayed gastric emptying with GLP-1 receptor agonists and tirzepatide. J. Clin. Endocrinol. Metab. 110 (1), 1–15. 10.1210/clinem/dgae719 39418085 PMC11651700

[B60] Jara M. Norlin J. Kjaer M. S. Almholt K. Bendtsen K. M. Bugianesi E. (2025). Modulation of metabolic, inflammatory and fibrotic pathways by semaglutide in metabolic dysfunction-associated steatohepatitis. Nat. Med. 31 (9), 3128–3140. 10.1038/s41591-025-03799-0 40691365 PMC12443624

[B61] Jastreboff A. M. Aronne L. J. Ahmad N. N. Wharton S. Connery L. Alves B. (2022). Tirzepatide once weekly for the treatment of obesity. N. Engl. J. Med. 387 (3), 205–216. 10.1056/NEJMoa2206038 35658024

[B62] Jee Y. M. Lee J. Y. Ryu T. (2025). Chronic inflammation and immune dysregulation in metabolic-dysfunction-associated steatotic liver disease progression: from steatosis to hepatocellular carcinoma. Biomedicines 13 (5), 1260. 10.3390/biomedicines13051260 40427086 PMC12109540

[B63] Jerlhag E. (2025). GLP-1 receptor agonists: promising therapeutic targets for alcohol use disorder. Endocrinology 166 (4), bqaf028. 10.1210/endocr/bqaf028 39980336 PMC11879929

[B64] Jones L. A. Brierley D. I. (2025). GLP-1 and the neurobiology of eating control: recent advances. Endocrinology 166 (2), bqae167. 10.1210/endocr/bqae167 39813121 PMC11745901

[B65] Kalligeros M. Vassilopoulos A. Vassilopoulos S. Victor D. W. Mylonakis E. Noureddin M. (2024). Prevalence of steatotic liver disease (MASLD, MetALD, and ALD) in the United States: NHANES 2017-2020. Clin. Gastroenterol. Hepatol. 22 (6), 1330–2 e4. 10.1016/j.cgh.2023.11.003 37949334

[B66] Kang L. Sebastian B. M. Pritchard M. T. Pratt B. T. Previs S. F. Nagy L. E. (2007). Chronic ethanol-induced insulin resistance is associated with macrophage infiltration into adipose tissue and altered expression of adipocytokines. Alcohol Clin. Exp. Res. 31 (9), 1581–1588. 10.1111/j.1530-0277.2007.00452.x 17624994

[B67] Kanwal F. Kramer J. R. Li L. Yang Y. X. Cao Y. Yu X. (2024). GLP-1 receptor agonists and risk for cirrhosis and related complications in patients with metabolic dysfunction-associated steatotic liver disease. JAMA Intern Med. 184 (11), 1314–1323. 10.1001/jamainternmed.2024.4661 39283612 PMC11406452

[B68] Karin M. Kim J. Y. (2025). MASH as an emerging cause of hepatocellular carcinoma: current knowledge and future perspectives. Mol. Oncol. 19 (2), 275–294. 10.1002/1878-0261.13685 38874196 PMC11793012

[B69] Kawaguchi T. Yoshida T. Harada M. Hisamoto T. Nagao Y. Ide T. (2004). Hepatitis C virus down-regulates insulin receptor substrates 1 and 2 through up-regulation of suppressor of cytokine signaling 3. Am. J. Pathol. 165 (5), 1499–1508. 10.1016/S0002-9440(10)63408-6 15509521 PMC1618659

[B70] Kharbanda K. K. Farokhnia M. Deschaine S. L. Bhargava R. Rodriguez-Flores M. Casey C. A. (2022). Role of the ghrelin system in alcohol use disorder and alcohol-associated liver disease: a narrative review. Alcohol Clin. Exp. Res. 46 (12), 2149–2159. 10.1111/acer.14967 36316764 PMC9772086

[B71] Kim K. H. Hong S. P. Kim K. Park M. J. Kim K. J. Cheong J. (2007). HCV core protein induces hepatic lipid accumulation by activating SREBP1 and PPARgamma. Biochem. Biophys. Res. Commun. 355 (4), 883–888. 10.1016/j.bbrc.2007.02.044 17331464

[B72] Klausen M. K. Jensen M. E. Moller M. Le Dous N. Jensen A. O. Zeeman V. A. (2022). Exenatide once weekly for alcohol use disorder investigated in a randomized, placebo-controlled clinical trial. JCI Insight 7 (19), e159863. 10.1172/jci.insight.159863 36066977 PMC9675448

[B73] Kojima M. Takahashi H. Kuwashiro T. Tanaka K. Mori H. Ozaki I. (2020). Glucagon-like Peptide-1 receptor agonist prevented the progression of hepatocellular carcinoma in a mouse model of nonalcoholic steatohepatitis. Int. J. Mol. Sci. 21 (16), 5722. 10.3390/ijms21165722 32785012 PMC7460814

[B74] Krause G. C. Lima K. G. Dias H. B. da Silva E. F. G. Haute G. V. Basso B. S. (2017). Liraglutide, a glucagon-like peptide-1 analog, induce autophagy and senescence in HepG2 cells. Eur. J. Pharmacol. 809, 32–41. 10.1016/j.ejphar.2017.05.015 28501576

[B75] Krause G. C. Lima K. G. Levorse V. Haute G. V. Gassen R. B. Garcia M. C. (2019). Exenatide induces autophagy and prevents the cell regrowth in HepG2 cells. EXCLI J. 18, 540–548. 10.17179/excli2019-1415 31611738 PMC6785771

[B76] Lawitz E. J. Fraessdorf M. Neff G. W. Schattenberg J. M. Noureddin M. Alkhouri N. (2024). Efficacy, tolerability and pharmacokinetics of survodutide, a glucagon/glucagon-like peptide-1 receptor dual agonist, in cirrhosis. J. Hepatol. 81 (5), 837–846. 10.1016/j.jhep.2024.06.003 38857788

[B77] Leal-Lassalle H. Estévez-Vázquez O. Cubero F. J. Nevzorova Y. A. (2025). Metabolic and alcohol-associated liver disease (MetALD): a representation of duality. NPJ Gut Liver 2 (1), 1. 10.1038/s44355-024-00014-8

[B78] Lee M. Y. Chen W. C. Hsu W. H. Chen S. C. Lee J. C. (2019). Liraglutide inhibits hepatitis C virus replication through an AMP activated protein kinase dependent mechanism. Int. J. Mol. Sci. 20 (18), 4569. 10.3390/ijms20184569 31540136 PMC6769880

[B79] Leith D. Lin Y. Y. Brennan P. (2024). Metabolic dysfunction-associated steatotic liver disease and type 2 diabetes: a deadly synergy. TouchREV Endocrinol. 20 (2), 5–9. 10.17925/EE.2024.20.2.2 39526052 PMC11548366

[B80] Leko A. H. Leggio L. (2024). Barriers to alcohol use disorder treatment in patients with alcohol-associated liver disease. Clin. Liver Dis. 28 (4), 779–791. 10.1016/j.cld.2024.06.012 39362721 PMC11458136

[B81] Li Y. Yang P. Ye J. Xu Q. Wu J. Wang Y. (2024). Updated mechanisms of MASLD pathogenesis. Lipids Health Dis. 23 (1), 117. 10.1186/s12944-024-02108-x 38649999 PMC11034170

[B82] Liu Y. Chen S. Zhen R. (2024). Effect of semaglutide on high-fat-diet-induced liver cancer in Obese mice. J. Proteome Res. 23 (2), 704–717. 10.1021/acs.jproteome.3c00498 38227547 PMC10846501

[B83] Liu J. Yang F. Gao B. Yang L. Cao Y. Zhou Y. (2025). Resmetirom, the first FDA-approved drug for MASH: from drug discovery and action mechanisms to clinical trials. Arch. Pharm. Res. 48 (11–12), 1299–1313. 10.1007/s12272-025-01574 41166060

[B84] Lu X. Xu C. Dong J. Zuo S. Zhang H. Jiang C. (2021). Liraglutide activates nature killer cell-mediated antitumor responses by inhibiting IL-6/STAT3 signaling in hepatocellular carcinoma. Transl. Oncol. 14 (1), 100872. 10.1016/j.tranon.2020.100872 32979685 PMC7516274

[B85] Ma Y. Wang J. Xiao W. Fan X. (2024). A review of MASLD-related hepatocellular carcinoma: progress in pathogenesis, early detection, and therapeutic interventions. Front. Med. (Lausanne) 11, 1410668. 10.3389/fmed.2024.1410668 38895182 PMC11184143

[B86] Mackowiak B. Fu Y. Maccioni L. Gao B. (2024). Alcohol associated liver disease. J. Clin. Invest. 134 (3), e176345. 10.1172/JCI176345 38299591 PMC10836812

[B87] Mahalingam S. Bellamkonda R. Arumugam M. K. Perumal S. K. Yoon J. Casey C. (2023). Glucagon-like peptide 1 receptor agonist, exendin-4, reduces alcohol-associated fatty liver disease. Biochem. Pharmacol. 213, 115613. 10.1016/j.bcp.2023.115613 37209859 PMC10351880

[B88] Mantovani A. Petracca G. Beatrice G. Csermely A. Lonardo A. Targher G. (2021). Glucagon-like Peptide-1 receptor agonists for treatment of nonalcoholic fatty liver disease and nonalcoholic steatohepatitis: an updated meta-analysis of randomized controlled trials. Metabolites 11 (2), 73. 10.3390/metabo11020073 33513761 PMC7911747

[B89] Marek G. W. Malhi H. (2024). MetALD: does it require a different therapeutic option? Hepatology 80 (6), 1424–1440. 10.1097/HEP.0000000000000935 38820071 PMC12172020

[B90] Miller D. M. McCauley K. F. Dunham-Snary K. J. (2025). Metabolic dysfunction-associated steatotic liver disease (MASLD): mechanisms, clinical implications and therapeutic advances. Endocrinol. Diabetes Metab. 8 (6), e70132. 10.1002/edm2.70132 41255342 PMC12627968

[B91] Mocciaro G. Capodici A. De Amicis R. (2025). GLP-1 receptor agonists induce loss of lean mass: so does caloric restriction. BMJ Nutr. Prev. Health 8 (1), e001206. 10.1136/bmjnph-2025-001206 40771503 PMC12322565

[B92] Mollerhoj M. B. Veidal S. S. Thrane K. T. Oro D. Overgaard A. Salinas C. G. (2022). Hepatoprotective effects of semaglutide, lanifibranor and dietary intervention in the GAN diet-induced obese and biopsy-confirmed mouse model of NASH. Clin. Transl. Sci. 15 (5), 1167–1186. 10.1111/cts.13235 35143711 PMC9099137

[B93] Muller T. D. Finan B. Bloom S. R. D'Alessio D. Drucker D. J. Flatt P. R. (2019). Glucagon-like peptide 1 (GLP-1). Mol. Metab. 30, 72–130. 10.1016/j.molmet.2019.09.010 31767182 PMC6812410

[B94] Nakamura T. Masuda A. Nakano D. Amano K. Sano T. Nakano M. (2025). Pathogenic mechanisms of metabolic dysfunction-associated steatotic liver disease (MASLD)-associated hepatocellular carcinoma. Cells 14 (6), 428. 10.3390/cells14060428 40136677 PMC11941585

[B95] Newsome P. N. Buchholtz K. Cusi K. Linder M. Okanoue T. Ratziu V. (2021). A placebo-controlled trial of subcutaneous semaglutide in nonalcoholic steatohepatitis. N. Engl. J. Med. 384 (12), 1113–1124. 10.1056/NEJMoa2028395 33185364

[B96] Nieto L. M. Martinez J. Narvaez S. I. Ko D. Kim D. H. Vega K. J. (2025). Glucagon-like Peptide-1 receptor agonists use does not increase the risk for acute pancreatitis and is associated with lower complications in patients with type 2 diabetes who develop acute pancreatitis: a multicenter analysis. Am. J. Gastroenterol. 121 (2), 424–431. 10.14309/ajg.0000000000003525 40358430

[B97] Ochoa-Allemant P. Serper M. Wang R. X. Tang H. Ghandour B. Khan S. (2025). Waitlisting and liver transplantation for MetALD in the United States: an analysis of the UNOS national registry. Hepatology 81 (2), 532–545. 10.1097/HEP.0000000000000914 38683569 PMC12036730

[B98] Osna N. A. Rasineni K. Ganesan M. Donohue T. M. Jr. Kharbanda K. K. (2022). Pathogenesis of alcohol-associated liver disease. J. Clin. Exp. Hepatol. 12 (6), 1492–1513. 10.1016/j.jceh.2022.05.004 36340300 PMC9630031

[B99] Pan C. W. Abboud Y. Chitnis A. Zhang W. Singal A. K. Wong R. J. (2025). Alcohol-associated liver disease mortality. JAMA Netw. Open 8 (6), e2514857. 10.1001/jamanetworkopen.2025.14857 40498484 PMC12159772

[B100] Pandey A. Patel K. V. Segar M. W. Ayers C. Linge J. Leinhard O. D. (2024). Effect of liraglutide on thigh muscle fat and muscle composition in adults with overweight or obesity: results from a randomized clinical trial. J. Cachexia Sarcopenia Muscle 15 (3), 1072–1083. 10.1002/jcsm.13445 38561962 PMC11154779

[B101] Pasta A. Facciorusso A. Plaz Torres M. C. Giannini E. G. Sacco R. (2025). Effects of glucagon-like PEPTIDE-1 receptor agonists on incidence of hepatocellular carcinoma and liver decompensation in patients with diabetes: a systematic review and META-analysis. Eur. J. Clin. Invest. 55 (6), e70000. 10.1111/eci.70000 39937048 PMC12066890

[B102] Patel R. Mueller M. (2025). Alcohol-associated liver disease. StatPearls. Treasure Isl. (FL). 31536239

[B103] Patel S. Blaney H. Nassar S. Singal A. K. (2025). GLP-1 receptor agonists and alcohol use disorder: a systematic review. Alcohol Alcohol 61 (1), agaf069. 10.1093/alcalc/agaf069 41273789

[B104] Pazienza V. Clement S. Pugnale P. Conzelman S. Foti M. Mangia A. (2007). The hepatitis C virus core protein of genotypes 3a and 1b downregulates insulin receptor substrate 1 through genotype-specific mechanisms. Hepatology 45 (5), 1164–1171. 10.1002/hep.21634 17465001

[B105] Perlemuter G. Sabile A. Letteron P. Vona G. Topilco A. Chretien Y. (2002). Hepatitis C virus core protein inhibits microsomal triglyceride transfer protein activity and very low density lipoprotein secretion: a model of viral-related steatosis. FASEB J. 16 (2), 185–194. 10.1096/fj.01-0396com 11818366

[B106] Petta S. Kim K. Targher G. Romeo S. Sookoian S. Zheng M. H. (2025). Focus on semaglutide 2.4 mg/week for the treatment of metabolic dysfunction-associated steatohepatitis. Liver Int. 45 (11), e70407. 10.1111/liv.70407 41144918 PMC12558666

[B107] Pieralice S. Amendolara R. Berna V. Manganaro G. Zurru A. D'Onofrio L. (2025). The emerging role of anti-hyperglycemic agents for the management of metabolic dysfunction-associated steatotic liver disease. Diabetes Metab. Syndr. Obes. 18, 2477–2491. 10.2147/DMSO.S528569 40718585 PMC12296652

[B108] Pinto L. C. Falcetta M. R. Rados D. V. Leitao C. B. Gross J. L. (2019). Glucagon-like peptide-1 receptor agonists and pancreatic cancer: a meta-analysis with trial sequential analysis. Sci. Rep. 9 (1), 2375. 10.1038/s41598-019-38956-2 30787365 PMC6382780

[B109] Probst L. Monnerat S. Vogt D. R. Lengsfeld S. Burkard T. Meienberg A. (2023). Effects of dulaglutide on alcohol consumption during smoking cessation. JCI Insight 8 (22), e170419. 10.1172/jci.insight.170419 37991022 PMC10721313

[B110] Przybyszewski E. M. Chung R. T. (2023). Unmet needs in the post-direct-acting antiviral era: hepatocarcinogenesis after hepatitis C virus eradication. J. Infect. Dis. 228 (Suppl. 3), S226–S231. 10.1093/infdis/jiac447 37703341 PMC10499186

[B111] Quddos F. Hubshman Z. Tegge A. Sane D. Marti E. Kablinger A. S. (2023). Semaglutide and Tirzepatide reduce alcohol consumption in individuals with obesity. Sci. Rep. 13 (1), 20998. 10.1038/s41598-023-48267-2 38017205 PMC10684505

[B112] Que Q. Guo X. Zhan L. Chen S. Zhang Z. Ni X. (2019). The GLP-1 agonist, liraglutide, ameliorates inflammation through the activation of the PKA/CREB pathway in a rat model of knee osteoarthritis. J. Inflamm. (Lond). 16, 13. 10.1186/s12950-019-0218-y 31182934 PMC6554939

[B113] Rizzo G. E. M. Cabibbo G. Craxi A. (2022). Hepatitis B virus-associated hepatocellular carcinoma. Viruses 14 (5), 986. 10.3390/v14050986 35632728 PMC9146458

[B114] Rajewski P. Ciescinski J. Rajewski P. Suwala S. Rajewska A. Potasz M. (2025). Dietary interventions and physical activity as crucial factors in the prevention and treatment of metabolic dysfunction-associated steatotic liver disease. Biomedicines 13 (1), 217. 10.3390/biomedicines13010217 39857800 PMC11760440

[B115] Ramaite F. T. Nkadimeng S. M. (2025). Targeting inflammatory pathways in hepatocellular carcinoma: recent developments. Discov. Oncol. 16 (1), 1174. 10.1007/s12672-025-03035-8 40544399 PMC12183140

[B116] Rasineni K. Casey C. A. (2012). Molecular mechanism of alcoholic fatty liver. Indian J. Pharmacol. 44 (3), 299–303. 10.4103/0253-7613.96297 22701235 PMC3371448

[B117] Rasineni K. Thomes P. G. Kubik J. L. Harris E. N. Kharbanda K. K. Casey C. A. (2019a). Chronic alcohol exposure alters circulating insulin and ghrelin levels: role of ghrelin in hepatic steatosis. Am. J. Physiol. Gastrointest. Liver Physiol. 316 (4), G453–G461. 10.1152/ajpgi.00334.2018 30702902 PMC6483023

[B118] Rasineni K. Kubik J. L. Casey C. A. Kharbanda K. K. (2019b). Inhibition of ghrelin activity by receptor antagonist [d-Lys-3] GHRP-6 attenuates alcohol-induced hepatic steatosis by regulating hepatic lipid metabolism. Biomolecules 9 (10), 517. 10.3390/biom9100517 31546643 PMC6843513

[B119] Riley D. R. Hydes T. Hernadez G. Zhao S. S. Alam U. Cuthbertson D. J. (2024). The synergistic impact of type 2 diabetes and MASLD on cardiovascular, liver, diabetes-related and cancer outcomes. Liver Int. 44 (10), 2538–2550. 10.1111/liv.16016 38949295

[B120] Sadagopan N. He A. R. (2024). Recent progress in systemic therapy for advanced hepatocellular carcinoma. Int. J. Mol. Sci. 25 (2), 1259. 10.3390/ijms25021259 38279258 PMC10816205

[B121] Salmon D. Bani-Sadr F. Loko M. A. Stitou H. Gervais A. Durant J. (2012). Insulin resistance is associated with a higher risk of hepatocellular carcinoma in cirrhotic HIV/HCV-co-infected patients: results from ANRS CO13 HEPAVIH. J. Hepatol. 56 (4), 862–868. 10.1016/j.jhep.2011.11.009 22173166

[B122] Sanyal A. J. Newsome P. N. Kliers I. Ostergaard L. H. Long M. T. Kjaer M. S. (2025). Phase 3 trial of semaglutide in metabolic dysfunction-associated steatohepatitis. N. Engl. J. Med. 392 (21), 2089–2099. 10.1056/NEJMoa2413258 40305708

[B123] Sargeant J. A. Henson J. King J. A. Yates T. Khunti K. Davies M. J. (2019). A review of the effects of glucagon-like Peptide-1 receptor agonists and sodium-glucose cotransporter 2 inhibitors on lean body mass in humans. Endocrinol. Metab. Seoul. 34 (3), 247–262. 10.3803/EnM.2019.34.3.247 31565876 PMC6769337

[B124] Schinzari V. Barnaba V. Piconese S. (2015). Chronic hepatitis B virus and hepatitis C virus infections and cancer: synergy between viral and host factors. Clin. Microbiol. Infect. 21 (11), 969–974. 10.1016/j.cmi.2015.06.026 26163104

[B125] Schonfeld M. O'Neil M. Villar M. T. Artigues A. Averilla J. Gunewardena S. (2021). A Western diet with alcohol in drinking water recapitulates features of alcohol-associated liver disease in mice. Alcohol Clin. Exp. Res. 45 (10), 1980–1993. 10.1111/acer.14700 34523155 PMC9006178

[B126] Sengupta S. Mellinger J. L. (2024). Preventive behavioral interventions for patients with steatotic liver disease. Clin. Liver Dis. Hob. 23 (1), e0202. 10.1097/CLD.0000000000000202 38872780 PMC11168848

[B127] Shabil M. Khatib M. N. Ballal S. Bansal P. Tomar B. S. Ashraf A. (2024). Risk of hepatocellular carcinoma with Glucagon-like Peptide-1 receptor agonist treatment in patients: a systematic review and meta-analysis. BMC Endocr. Disord. 24 (1), 246. 10.1186/s12902-024-01775-2 39551761 PMC11571652

[B128] Shantaram D. Rima X. Y. Bradley D. Liu J. Z. Wright V. P. Amari A. (2025). The GLP-1 receptor agonist dulaglutide attenuates hepatic steatosis in obesity *via* a weight-independent mechanism. Diabetes 74 (9), 1512–1524. 10.2337/db24-0861 40663700 PMC12365420

[B129] Sharma S. Mells J. E. Fu P. P. Saxena N. K. Anania F. A. (2011). GLP-1 analogs reduce hepatocyte steatosis and improve survival by enhancing the unfolded protein response and promoting macroautophagy. PLoS One 6 (9), e25269. 10.1371/journal.pone.0025269 21957486 PMC3177901

[B130] Shi Y. Taherifard E. Saeed A. Saeed A. (2024). MASLD-related HCC: a comprehensive review of the trends, pathophysiology, tumor microenvironment, surveillance, and treatment options. Curr. Issues Mol. Biol. 46 (6), 5965–5983. 10.3390/cimb46060356 38921027 PMC11202630

[B131] Shin H. J. Park Y. H. Kim S. U. Moon H. B. Park D. S. Han Y. H. (2011). Hepatitis B virus X protein regulates hepatic glucose homeostasis *via* activation of inducible nitric oxide synthase. J. Biol. Chem. 286 (34), 29872–29881. 10.1074/jbc.M111.259978 21690090 PMC3191028

[B132] Singal A. K. Leggio L. (2025). GLP-1 receptor agonists in alcohol use disorder and alcohol-associated liver disease. Lancet Gastroenterol. Hepatol. 10 (8), 707–709. 10.1016/S2468-1253(25)00134-7 40482663

[B133] Skibicka K. P. (2013). The central GLP-1: implications for food and drug reward. Front. Neurosci. 7, 181. 10.3389/fnins.2013.00181 24133407 PMC3796262

[B134] Sodhi M. Rezaeianzadeh R. Kezouh A. Etminan M. (2023). Risk of gastrointestinal adverse events associated with glucagon-like Peptide-1 receptor agonists for weight loss. JAMA 330 (18), 1795–1797. 10.1001/jama.2023.19574 37796527 PMC10557026

[B135] Stefan N. Yki-Jarvinen H. Neuschwander-Tetri B. A. (2025). Metabolic dysfunction-associated steatotic liver disease: heterogeneous pathomechanisms and effectiveness of metabolism-based treatment. Lancet Diabetes Endocrinol. 13 (2), 134–148. 10.1016/S2213-8587(24)00318-8 39681121

[B136] Storgaard H. Cold F. Gluud L. L. Vilsboll T. Knop F. K. (2017). Glucagon-like peptide-1 receptor agonists and risk of acute pancreatitis in patients with type 2 diabetes. Diabetes Obes. Metab. 19 (6), 906–908. 10.1111/dom.12885 28105738

[B137] Suhail M. Sohrab S. S. Kamal M. A. Azhar E. I. (2022). Role of hepatitis c virus in hepatocellular carcinoma and neurological disorders: an overview. Front. Oncol. 12, 913231. 10.3389/fonc.2022.913231 35965577 PMC9372299

[B138] Tamilwanan S. Aziz Z. Rong L. Y. Bitar A. N. Zarzour R. H. A. Alshehade S. A. (2025). Efficacy of GLP-1 receptor agonists and dual GLP-1/GIP receptor agonists in managing MALFD: a meta-analysis of randomized controlled trials. BMC Gastroenterol. 25 (1), 765. 10.1186/s12876-025-04358-0 41146009 PMC12560356

[B139] Tantu M. T. Farhana F. Z. Haque F. Koo K. M. Qiao L. Ross A. G. (2025). Pathophysiology, noninvasive diagnostics and emerging personalized treatments for metabolic associated liver diseases. Gut Liver 2 (1), 18. 10.1038/s44355-025-00030-2

[B140] Vendrell J. El Bekay R. Peral B. Garcia-Fuentes E. Megia A. Macias-Gonzalez M. (2011). Study of the potential association of adipose tissue GLP-1 receptor with obesity and insulin resistance. Endocrinology 152 (11), 4072–4079. 10.1210/en.2011-1070 21862620

[B141] Wan S. Sun H. (2019). Glucagon-like peptide-1 modulates RAW264.7 macrophage polarization by interfering with the JNK/STAT3 signaling pathway. Exp. Ther. Med. 17 (5), 3573–3579. 10.3892/etm.2019.7347 30988739 PMC6447820

[B142] Wang M. W. Lu L. G. (2025). Current status of glucagon-like Peptide-1 receptor agonists in metabolic dysfunction-associated steatotic liver disease: a clinical perspective. J. Clin. Transl. Hepatol. 13 (1), 47–61. 10.14218/JCTH.2024.00271 39801787 PMC11712088

[B143] Wang H. Zhang J. (2023). The glucose metabolic reprogramming in hepatitis B virus infection and hepatitis B virus associated diseases. J. Gastroenterol. Hepatol. 38 (11), 1886–1891. 10.1111/jgh.16340 37654246

[B144] Wang C. Li Q. Wang W. Guo L. Guo C. Sun Y. (2015). GLP-1 contributes to increases in PGC-1alpha expression by downregulating miR-23a to reduce apoptosis. Biochem. Biophys. Res. Commun. 466 (1), 33–39. 10.1016/j.bbrc.2015.08.092 26315270

[B145] Wang A. Li T. An P. Yan W. Zheng H. Wang B. (2017). Exendin-4 upregulates adiponectin level in adipocytes *via* Sirt1/Foxo-1 signaling pathway. PLoS One 12 (1), e0169469. 10.1371/journal.pone.0169469 28122026 PMC5266308

[B146] Wang J. Lu H. Li Q. (2024a). Hepatic macrophage niche: a bridge between HBV-mediated metabolic changes with intrahepatic inflammation. Front. Immunol. 15, 1414594. 10.3389/fimmu.2024.1414594 39091506 PMC11291371

[B147] Wang L. Berger N. A. Kaelber D. C. Xu R. (2024b). Association of GLP-1 receptor agonists and hepatocellular carcinoma incidence and hepatic decompensation in patients with type 2 diabetes. Gastroenterology 167 (4), 689–703. 10.1053/j.gastro.2024.04.029 38692395 PMC12294230

[B148] Weghuber D. Barrett T. Barrientos-Perez M. Gies I. Hesse D. Jeppesen O. K. (2022). Once-weekly semaglutide in adolescents with obesity. N. Engl. J. Med. 387 (24), 2245–2257. 10.1056/NEJMoa2208601 36322838 PMC9997064

[B149] Wei X. Shi X. Zhong W. Zhao Y. Tang Y. Sun W. (2013). Chronic alcohol exposure disturbs lipid homeostasis at the adipose tissue-liver axis in mice: analysis of triacylglycerols using high-resolution mass spectrometry in combination with *in vivo* metabolite deuterium labeling. PloS One 8 (2), e55382. 10.1371/journal.pone.0055382 23405143 PMC3566154

[B150] Wei J. C. Wu C. N. Cheng W. C. (2025). Comments on association of GLP-1 receptor agonists and hepatocellular carcinoma incidence and hepatic decompensation in patients with type 2 diabetes. Gastroenterology 168 (2), 424. 10.1053/j.gastro.2024.09.039 39426486

[B151] Wharton S. Calanna S. Davies M. Dicker D. Goldman B. Lingvay I. (2022). Gastrointestinal tolerability of once-weekly semaglutide 2.4 mg in adults with overweight or obesity, and the relationship between gastrointestinal adverse events and weight loss. Diabetes Obes. Metab. 24 (1), 94–105. 10.1111/dom.14551 34514682 PMC9293236

[B152] Wickramarachchi N. Sobero A. Prevallet A. Dorgalli C. Shahbaz O. (2025). S2830 alcohol use reduction with GLP-1 agonist use. Official Journal Am. Coll. Gastroenterology | ACG. 120 (10S2), S608. 10.14309/01.ajg.0001138780.07976.e4

[B153] Wilding J. P. H. Batterham R. L. Calanna S. Davies M. Van Gaal L. F. Lingvay I. (2021). Once-weekly semaglutide in adults with overweight or obesity. N. Engl. J. Med. 384 (11), 989–1002. 10.1056/NEJMoa2032183 33567185

[B154] Yan C. Hu W. Tu J. Li J. Liang Q. Han S. (2023). Pathogenic mechanisms and regulatory factors involved in alcoholic liver disease. J. Transl. Med. 21 (1), 300. 10.1186/s12967-023-04166-8 37143126 PMC10158301

[B155] Yang F. Yan S. He Y. Wang F. Song S. Guo Y. (2008). Expression of hepatitis B virus proteins in transgenic mice alters lipid metabolism and induces oxidative stress in the liver. J. Hepatol. 48 (1), 12–19. 10.1016/j.jhep.2007.06.021 18037187

[B156] Younossi Z. M. Stepanova M. Ong J. Trimble G. AlQahtani S. Younossi I. (2021). Nonalcoholic steatohepatitis is the Most rapidly increasing indication for liver transplantation in the United States. Clin. Gastroenterol. Hepatol. 19 (3), 580–9 e5. 10.1016/j.cgh.2020.05.064 32531342

[B157] Yu X. Bian X. Zhang H. Yang S. Cui D. Su Z. (2023). Liraglutide ameliorates hepatic steatosis *via* retinoic acid receptor-related orphan receptor alpha-mediated autophagy pathway. IUBMB Life 75 (10), 856–867. 10.1002/iub.2760 37310057

[B158] Zhang X. Cao C. Zheng F. Liu C. Tian X. (2025). Therapeutic potential of GLP-1 receptor agonists in diabetes and cardiovascular disease: mechanisms and clinical implications. Cardiovasc Drugs Ther. 40 (1), 287–301. 10.1007/s10557-025-07670-9 39832069 PMC12872670

[B159] Zhao H. Wang L. Wei R. Xiu D. Tao M. Ke J. (2014). Activation of glucagon-like peptide-1 receptor inhibits tumourigenicity and metastasis of human pancreatic cancer cells *via* PI3K/Akt pathway. Diabetes Obes. Metab. 16 (9), 850–860. 10.1111/dom.12291 24641303

[B160] Zhong W. Zhao Y. Tang Y. Wei X. Shi X. Sun W. (2012). Chronic alcohol exposure stimulates adipose tissue lipolysis in mice: role of reverse triglyceride transport in the pathogenesis of alcoholic steatosis. Am. J. Pathol. 180 (3), 998–1007. 10.1016/j.ajpath.2011.11.017 22234172 PMC3349880

[B161] Zhou M. Mok M. T. Sun H. Chan A. W. Huang Y. Cheng A. S. (2017). The anti-diabetic drug exenatide, a glucagon-like peptide-1 receptor agonist, counteracts hepatocarcinogenesis through cAMP-PKA-EGFR-STAT3 axis. Oncogene 36 (29), 4135–4149. 10.1038/onc.2017.38 28319060

